# GLP1 receptor agonists and SGLT2 inhibitors for the prevention or delay of type 2 diabetes mellitus onset: a systematic review and meta-analysis

**DOI:** 10.3389/fendo.2025.1627909

**Published:** 2025-10-23

**Authors:** Farah Ghobar, Ali Tarhini, Zeinab Osman, Sergio Sbeih, Ralph Abou Ghayda, Patena Matar, Gaelle Haddad, Amjad Kanaan, Assaad Eid, Sami Azar, Hilda E. Ghadieh, Frederic Harb

**Affiliations:** ^1^ Faculty of Medicine and Medical Sciences, University of Balamand, Kalhat, Lebanon; ^2^ AUB Diabetes, American University of Beirut, Beirut, Lebanon; ^3^ Department of Anatomy, Cell Biology, and Physiological Sciences, Faculty of Medicine, American University of Beirut, Beirut, Lebanon

**Keywords:** type two diabetes mellitus (T2DM), sodium-glucose transport protein 2 (SGLT-2), Glucagon-like peptide-1 (GLP-1), prediabetes, HbA1c, fasting glucose, fasting insulin

## Abstract

**Background:**

SGLT-2 inhibitors (SGLT-2i) and GLP-1 receptor agonists (GLP-1 RA) are two widely used classes of medications in the treatment of diabetes, each demonstrating significant efficacy and adoption. These medications have shown promising results in glycemic control and offer additional health benefits such as weight loss and cardiovascular protection. The objective of our meta-analysis is to systematically assess the effectiveness of SGLT-2 inhibitors and GLP-1 receptor agonists in slowing down or preventing the progression of prediabetic patients into diabetics. By synthesizing the existing evidence, we aim to determine whether early intervention with these medications can effectively mitigate the risk of developing diabetes in prediabetic individuals. This analysis will provide critical insights into their comparative effectiveness and inform clinical decision-making for early diabetes prevention strategies.

**Methods:**

In this meta-analysis we primarily focused on randomized control trials (RCTs) that included the use of either SGLT-2 inhibitors, GLP-1 receptor agonists, or both. The database search was conducted using PubMed, Embase, and Cochrane. Statistical analysis was performed using IBM SPSS, and the results were reported with a 95% confidence interval (CI).

**Results:**

The meta-analysis included 14 studies with the majority being double blinded (13/14). Effective randomization was evident from balanced baseline characteristics between treatment and control groups. Both SGLT-2 inhibitors and GLP-1 receptor agonists demonstrated significant reductions in body weight when given individually. This effect is also amplified when given as a combination therapy (SMD: -23, 95% CI: [-27.9, -18.10]). Also, fasting plasma glucose levels decreased in patients receiving treatment (SMD: -5.40, 95% CI: [-10.70, 2.24]) compared to control groups. Moreover, HbA1c levels were assessed in seven studies, where significant reductions in treatment groups were reported with a standardized mean difference of -6.95 (95% CI: [-14.24, 2.98], p-value= 0.06) for the overall effect size. Furthermore, three studies showed that SGLT-2 inhibitors reduced diabetes mellitus (DM) onset, though statistical significance was not achieved (p-value = 0.08, SMD: -2.21, CI: [-5.11, 0.69]). Finally, no significant change in fasting insulin levels was noticed with an overall SMD of -1.74 (95% CI: [-6.84, 3.37]), which was also not statistically significant (p-value: 0.55). These findings highlight the efficacy of SGLT-2 inhibitors and GLP-1 receptor agonists in reducing HbA1C, fasting blood glucose, and body weight, while also potentially delaying the progression to type 2 diabetes mellitus in prediabetic patients.

**Conclusion:**

Early medical intervention at the prediabetic stage with SGLT-2i or GLP-1 RA shows potential in modifying progression to the onset of T2DM and its adverse effects. However, more studies are needed to reliably assess which of the two yields better results and further investigate the potential of combination therapy.

**Systematic review registration:**

https://www.crd.york.ac.uk/prospero/, identifier CRD42024565439.

## 1 Introduction

Type 2 diabetes mellitus (T2DM) is defined as the lack of uptake of sugar into the muscles, fat and liver in response to their resistance to insulin. This insulin resistance slows the metabolism of sugar in the cells, leading to their accumulation in the blood causing hyperglycemia. With prolonged impairments in insulin sensitivity, a subsequent failure of pancreatic islets will occur as a result of its attempts to compensate for this persistence in hyperglycemia by maintaining the production of insulin (1). The diagnosis of diabetes according to the American Diabetes Association can be done by using the glycated hemoglobin test, known as HbA1c. HbA1c levels greater than 6.5% is diagnostic for diabetes, while HbA1C below 5.7% excludes diabetes. However, the population whose HbA1c levels fall between 5.7% and 6.5% are at a stage known as “prediabetes”. This stage is described as an intermediate population that are at risk of developing diabetes later in life if no interventions are implied (2). This classification could help identify and screen individuals, enabling the selection of a target population for early intervention. This approach aims to prevent diabetes from developing and delay the onset of its complications. Diabetes mellitus can lead to both microvascular and macrovascular complications. Microvascular complications include retinopathy as well as nephropathy and neuropathy, while macrovascular complications include ischemic heart disease, peripheral vascular disease and cerebrovascular diseases. Both types of complications can result in organ and tissue damage, which was reported in almost one third to half of people with diabetes (3).

Recently, a combination of many anti-hyperglycemics is being used in the treatment and prevention of type 2 diabetes’ complications, yet few drugs are studied for their efficacy when prescribed for prediabetics (2). GLP-1 receptor agonists and SGLT-2 inhibitors are used as efficacious drugs for treatment of type two diabetes mellitus, offering positive benefits on both weight loss and blood pressure, with limited risk for hypoglycemia. Both of those agents also play an essential role in protection against major cardiovascular events in patients with known atherosclerotic cardiovascular disease, as well as reducing the risk of hospitalization for heart failure and all-cause mortality (4). SGLT-2 inhibitors act by blocking the reuptake of glucose, mainly in the proximal convoluted tubules where they are located. The Sodium-Glucose Transport Protein 2 uses the high energy yielded from the sodium gradient maintained by the Na^+^/K^+^ ATPase pump to absorb glucose back into the bloodstream. Blocking this receptor enhances glucose excretion in the urine, a process known as glucosuria (5). Glucagon-like peptide-1 (GLP-1) released from gut enteroendocrine cells controls meal-related glycemic excursions through augmentation of insulin and inhibition of glucagon secretion. It also inhibits gastric emptying and decreases food intake (6). Hence, GLP-1 receptor agonists will cause a glucose dependent increase in insulin while decreasing glucagon secretion and slowing stomach emptying.

Although SGLT-2 inhibitors and GLP-1 receptor agonists are crucial drugs in the management of type 2 diabetes, their potential role in delaying the progression of diabetes in prediabetic patients is yet to be discovered. This will be crucial in determining the approach that should be made by clinicians with these patients. It would also provide a cohesive decision-making guideline for physicians to follow in the race to decrease diabetes incidence. Therefore, this meta-analysis aims to assess the role of SGLT-2 inhibitors and GLP-1 receptor agonists in the prevention or delay of type 2 diabetes mellitus in patients classified as prediabetics.

## 2 Methodology

### 2.1 Eligibility criteria

In our meta-analysis, we applied specific inclusion and exclusion criteria to select our studies in a focused and reliable manner. The inclusion criteria primarily were limited to randomized controlled trials (RCTs) involving individuals who were either prediabetic or non-diabetic and treated with either SGLT-2 inhibitors, GLP-1 receptor agonists, or both. Within these findings, our primary outcome measure focused on HbA1c changes. This was then followed by identifying overlaps in secondary outcome measurements which included time to onset of Diabetes Mellitus as well as basic glycemic outcomes such as fasting plasma glucose, fasting insulin, and body weight changes, which were then integrated in our data. Exclusion criteria encompassed studies that involved diabetic patients as the treatment group, studies investigating the side effects of these drugs on heart and kidney function, follow-up trials, non-randomized controlled trials (RCTs) and studies not involving control groups (eg. comparing different treatment groups).

### 2.2 Sources

Our data was compiled from multiple sources. Databases from PubMed, Embase and Cochrane were extensively searched, primarily in search of clinical trials. This was achieved by applying filters to limit our search to ‘Randomized Controlled Trials (RCTs)’ where applicable. In studies registered in clinicaltrials.gov, available data was accessed for thorough screening and extraction of accurate results. Each database was analyzed and filtered through by at least 2 authors to ensure in depth analysis. To guarantee precision, the same keywords and their combinations were repeated across the different databases.

### 2.3 Search strategy

Our search strategy focused on identifying a comprehensive range of studies primarily investigating the impact of SGLT-2i and/or GLP-1 RA drugs on prediabetic or non-diabetic, healthy patients, with a specific focus on those assessing their effect on glycemic measures and diabetes onset. We utilized targeted keywords including “SGLT2 inhibitors”, “SGLT2”, “prediabetes”, “GLP1 agonist”, “GLP1”, “delay of diabetes”, “non-diabetic”, “T2DM” and “prevention” to pinpoint relevant literature in the databases. Some combinations of these include “(SGLT2) AND (prediabetes)”, “(GLP1) AND (prediabetes)”, “(SGLT2 inhibitors) AND (T2DM) AND (prevention)”, “(SGLT2) AND (GLP1) AND (prediabetes)”, and so forth. In addition, to further exclude studies exploring topics that deviate from our primary focus, our keywords included “NOT (heart)”, “NOT (kidney)”. Thus, the search string that we applied is as follows: (“GLP1 receptor agonists” OR “GLP-1 receptor agonists” OR “glucagon-like peptide-1 receptor agonists”) AND (“SGLT2 inhibitors” OR “SGLT-2 inhibitors” OR “sodium-glucose cotransporter 2 inhibitors”) AND (“Type 2 diabetes mellitus” OR “T2DM” OR “type 2 diabetes”) AND (“prevention” OR “delay” OR “onset”) AND (“efficacy” OR “effectiveness”) AND (“safety” OR “adverse effects”) AND (“patient characteristics” OR “age” OR “BMI” OR “comorbidities”) AND (“dosage” OR “dose” OR “treatment duration”) AND (“combination therapy” OR “synergistic effects”).

### 2.4 Selection process

**Figure 1:**
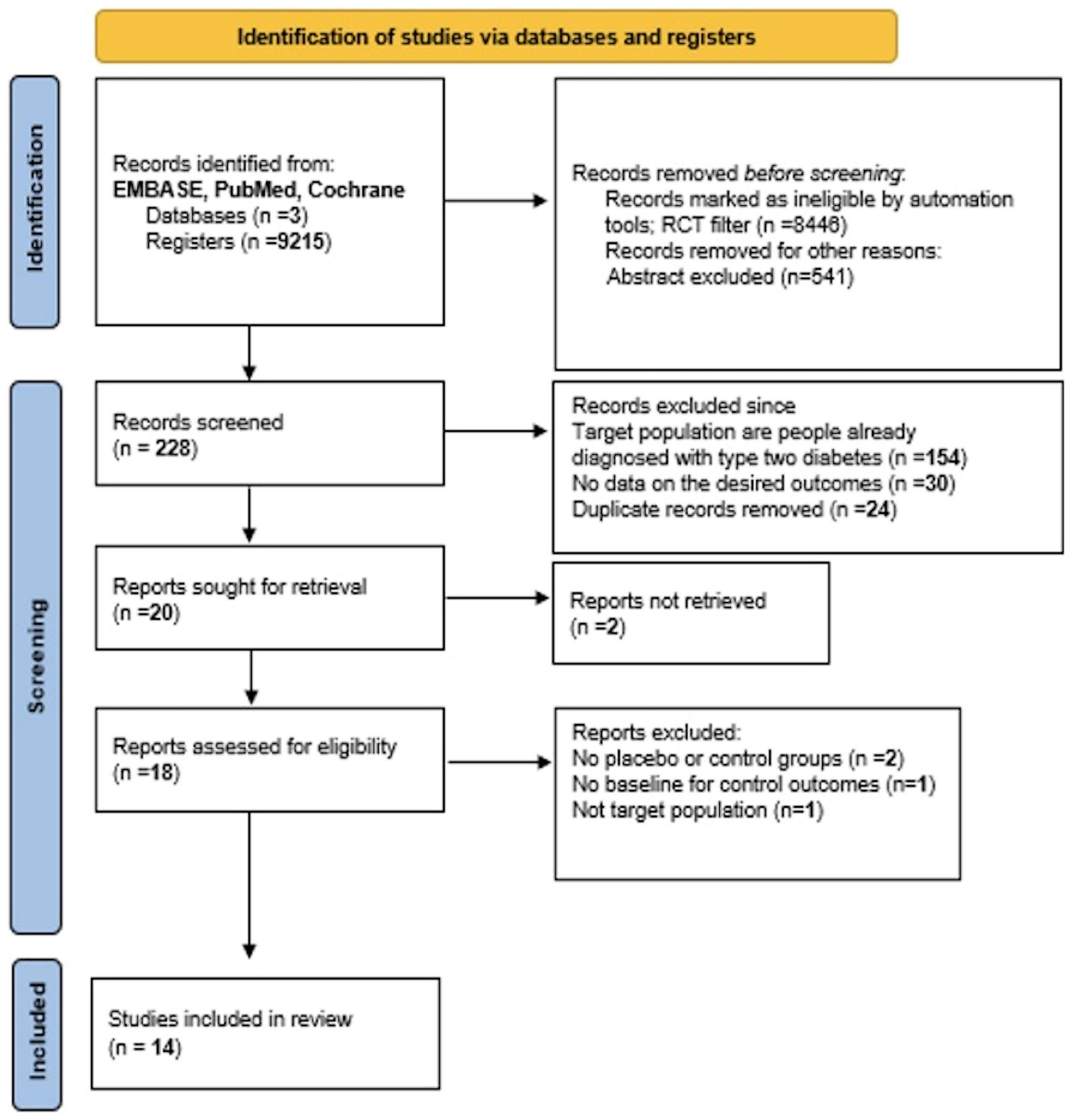
PRISMA flowchart of the included studies.

In our selection, we used the PRISMA model to guide our process (see **Figure 1**). Our search was categorized into three treatment groups: studies focusing solely on SGLT-2i, on GLP-1 RA, or on the combination of the two. Our initial screening method was done through the titles and abstracts of papers identified through our database search after applying an automated RCT filter. Papers that appeared to meet our eligibility criteria or warranted closer scrutiny for inclusion/exclusion were selected for further review and compiled into a word document. These were then organized into an excel sheet and separated by database, which allowed any duplicates to be easily identified and removed. The remaining studies were then combined into one sheet and divided evenly among 7 team members.

Each member independently assessed their studies more thoroughly, strictly adhering to our eligibility criteria. Each study was dissected down into its authors’ names, publication year, diagnostic criteria, intervention groups, dose and duration of treatment, and all reported outcomes. To enhance accuracy, every study underwent review by at least two authors.

### 2.5 Data collection

During data collection, our team meticulously reviewed each study, extracting essential information from the baseline characteristics table, results section, and supplementary materials as required. We gathered details including author names, publication dates, sample sizes, diagnostic criteria, intervention specifics (such as dose and treatment duration), and pertinent outcomes. This information was consolidated into a shared spreadsheet, with any necessary refinements collaboratively discussed and implemented among team members.

### 2.6 Data items

In our meta-analysis, the primary outcome was HbA1c levels. This outcome was consistently reported across nine papers, allowing for meaningful comparisons. Additionally, we identified several secondary outcomes common among the studies that further enriched our analysis of glycemic measures. These outcomes included incidence of diabetes onset, fasting insulin, fasting plasma glucose and body weight. Each outcome was highlighted distinctly to differentiate studies that shared similar measures. We then divided the studies based on the outcomes they reported.

### 2.7 Effect measures

In this meta-analysis, our main focus was to analyze the different outcomes of each study by calculating the mean difference (MD) and standard deviation (SD), and to check if those values were statistically significant through assessing their respective p-values. The primary outcome that is relevant in most of the included studies was HbA1c, which assesses the blood glucose control in prediabetic patients after administering SGLT-2i or GLP-1 RA compared to control. The mean difference in each study was calculated by subtracting the average HbA1c levels in patients from their correlated baseline measurements after the end of the exposure in each group. The same was done for all other measurements, including body weight, fasting plasma glucose, fasting insulin and DM onset.

### 2.8 Synthesis methods

Firstly, all articles that contained either SGLT-2 inhibitors or GLP-1 receptor agonists or both, with a mention of prediabetes or delay of diabetes, were selected and divided based on the treatment group.

The mean difference with standard deviation and 95% CI data was extracted by authors from the tables in each article or in some instances, either from supplementary data or clinicaltrials.gov. Papers were finally selected based on inclusion and exclusion criteria, and for each outcome a set of articles that included the specific outcome were grouped.

IBM SPSS Statistics 30.0.0.0 for Windows was used for statistical analysis. Authors chose Raw data for more accurate analysis. Random model effect was used to account for any variability in populations, interventions or methods. Inverse - Variance was chosen as the weighting method, and Restricted maximum likelihood (REML) was used for the variance estimator. Concerning the summary effect confidence interval, we used Truncated Hartung-Knapp-Sidik-Jonkman to explore the causes of statistical heterogeneity, and Egger’s regression base test was conducted.

### 2.9 Reporting bias assessment

**Figure 2:**
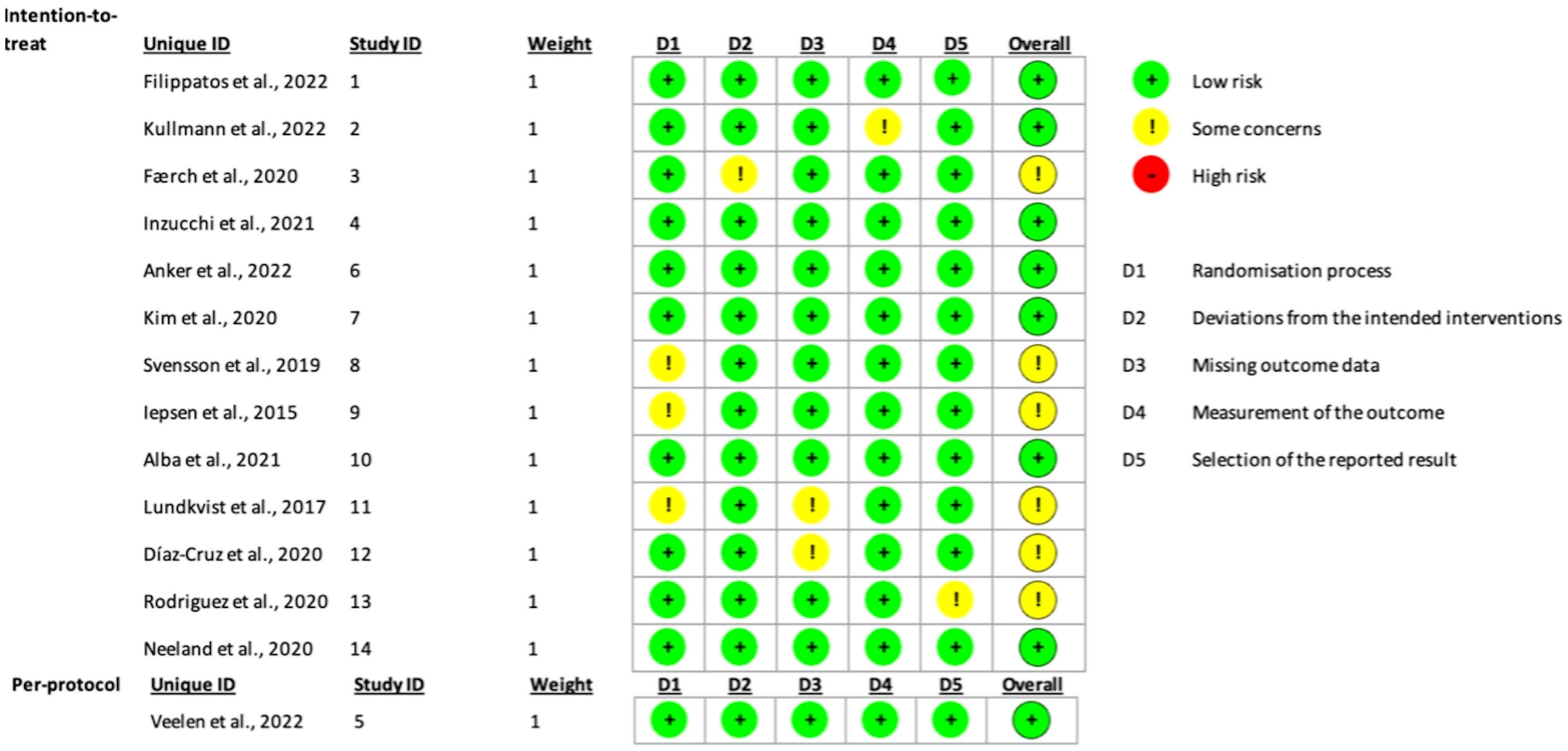
Traffic light plot used to display the risk of bias of the included studies based on Cochrane’s RoB 2.0 tool.

In order to assess the degree of bias in our selected articles, the authors of this meta-analysis evenly distributed the articles among themselves. As a result, the authors were subsequently paired into groups of two, each reviewing two articles independently for a total of 14 articles. In conducting our risk of bias, we used the Cochrane Collaboration Risk tool, which evaluates several domains including selection bias, performance bias, and reporting bias. Each of the authors individually reviewed and assessed the bias in their assigned articles. Afterward, both authors in each pair compared their evaluations to ensure consistency. In cases where their assessments differed, the authors engaged in discussions to explain their reasoning behind their judgment and reach a mutual agreement on the degree of bias in their articles. The tool used includes five distinct domains, each featuring specific questions to gauge the overall risk of bias in the article. Questions in domain one assessed the randomization process for selecting the subjects in each trial, while domain two reviewed if there were any deviations from the intended interventions. Domains three and four assessed the methods of measuring the outcomes and if there were any missing outcomes at the end of the trial. And finally, domain five assessed the selection of the reported results in each study. Based on the results of those five different domains, the overall bias was then calculated. Following the given algorithm and the assessors’ final judgment, none of the articles were found to have a high risk of bias, with a total of six articles identified as having “some concerns” regarding bias. The remaining articles were determined to have a low risk of bias. This approach provided a solid basis for assessing the validity and quality of the studies included in our systematic review. It also solidified the transparency and reliability of our meta-analysis. **Figure 2** provides a summary of the bias assessment outcomes.

### 2.10 Certainty assessment

The assessment was based on GRADE approach (Grading of Recommendations Assessment, Development, Evaluation):

1- Risk of Bias: The Cochrane Collaboration Risk tool was used by each author for the identified articles. Each article was reviewed twice, and bias was assessed as either having low, high, or some concerns. The CI was high enough which made us sure that the true effect size fell within the interval that we were looking at which was low bias.
2- Heterogeneity: Considerable heterogeneity was established in our study as we used the homogeneity/heterogeneity in the SPSS forest plot for HbA1c levels, DM onset, fasting insulin, fasting plasma glucose, and body weight, and for all these outcomes, I-squared fell between 99-100%. Each article used had different intervention protocols regarding dosage, length of assessment and either the usage of SGLT-2i or GLP-1 RA. All the studies were RCTs.
3- Imprecision: to reduce the chance of imprecision in our studies, the trim and fill feature used in SPSS studied the confidence interval, and the different outcomes obtained were adjusted to reach a higher certainty level.
4- Publication Bias: The Cochrane Risk of Bias 2.0 tool was used to assess the risk of bias in all studies included. This tool evaluates random sequence generation, allocation concealment, blinding of participants and personnel, incomplete outcome data, selective reporting, and other sources of bias. Assessments of risk of bias and quality were conducted independently by two reviewers, with discrepancies resolved through discussion or consultation with a third reviewer. The assessments are used to inform the interpretation of the findings and the strength of the conclusions drawn from the meta-analysis. The bias analysis results are summarized in **Figure 1**.

## 3 Results

### 3.1 Study selection

PRISMA 2020 flow diagram for new systematic reviews (see **Figure 1**) indicates the included searches of databases and registers only. Four studies were excluded from our meta-analysis, and the reasons for exclusion are as follow:

- The NCT02411825 trial was excluded since in its second part, patients with type two diabetes were included which is an exclusion criterion for our meta-analysis.
- The Gillani et al. (7) and Silver et al. (8) studies were excluded since there was no control group taking a placebo to be compared with the treatment group.
- Edwards et al. (9) study was also excluded since there was no given baseline for the control outcomes to be compared with the baseline of the patients who took the drug.

### 3.2 Characteristics of included studies

The characteristics of the included studies are summarized in **Table 1**, providing an overview of study design, sample size, type of drug and interventions.

**Table 1:** Characteristics of included studies.

Study	Year	Sample size (treatment/control)	Study design	Type of drug	Intervention time
(10) Filippatos et al.,	2022	2997/2991	International, phase III, double-blinded, parallel-group, placebo-controlled trial	Empagliflozin	52 weeks
(11) Kullmann et al.,	2022	19/21	randomized, double-blind, placebo-controlled, phase II trial	Empagliflozin	8 weeks
(12) Færch et al.,	2020	28/28	randomized, controlled, parallel, multi-arm, open-label, non-blinded trial	Dapagliflozin	13 weeks
(13) Inzucchi et al,	2021	1298/1307	multinational randomized, double-blind, placebo-controlled trial	Dapagliflozin	18.2 months
(14) Veelen et al,	2022	6/8	double-blinded, randomized, placebo-controlled, cross-over design	Dapagliflozin	2 weeks
(15) Anker et at	2022	632/636	double-blinded, randomized, placebo-controlled trial	Empagliflozin	1040 days
(16) Sun H. Kim et al,	2020	16/19	A double-blind, randomized clinical trial with a placebo control group	Liraglutide	14 weeks
(17) C. K. Svensson et al,	2019	46/50	randomized, double-blinded, placebo-controlled trial	Liraglutide	16 weeks
(18) EW Iepsen et al,	2015	27/25	A double-blind, randomized clinical trial with a placebo control group	Liraglutide	8 weeks
(19) Alba et al.,	2021	108/60	randomized, double-blind, placebo-controlled, open-label active-controlled, parallel-group, 5-arm, multicentre	Liraglutide	26 weeks
(20) Per Lundkvist et al,	2017	23/20	single-centre, randomized, parallel-group, double-blind, placebo-controlled phase IIa study	Dapagliflozin & Exenatide	24 weeks
(21) Díaz-Cruz et al,	2020	15/15	A double-blind, randomized clinical trial with a placebo control group	Dapagliflozin	12 weeks
(22) Rodríguez et al.,	2020	12/12	A randomized, double-blind, placebo-controlled clinical trial	Dapagliflozin	12 weeks
(23) Ian J. Neeland et al,	2020	18/17	A randomized, double-blind, placebo-controlled trial	Empagliflozin	12 weeks

### 3.3 Results of outcomes in respective individual studies

Starting with the first study used in our meta-analysis, it reported the difference in time to DM onset after treatment with empagliflozin (10) compared to a placebo (MD = 6.12, SD = 0.02), but those results were not statistically significant (p-value=0.13). It also reported how empagliflozin affected body weight, where a significant decrease was noted both in patients with and without diabetes (MD= -2.52 Kg, SD = 0.35) which was statistically significant (p- value<0.001) compared to the placebo group. To note that this study also showed a slight decrease in HbA1c levels but in diabetic patients only, whilst it had no noticeable effect on the pre-diabetic patients.

The second study used in our meta-analysis reported HbA1c levels after treatment with empagliflozin (11) and was analyzed using a random-effects model. However, the decrease after treatment was not very considerable (MD= -0.07, SD = 0.29, p-value=0.76). For fasting insulin, empagliflozin also showed no significant effect (MD= -1.15, SD = 0.14, p-value= 0.73). For fasting plasma glucose, treatment showed a statistically significant decrease compared to placebo (MD= -0.49mmol/l, SD = 0.03, p-value=0.03). This study also showed that body weight and BMI did not change (p-value>0.1), yet there was a decrease in the total amount of adipose tissue mass.

The third study by Færch et al. (12) reported data that are estimated to mean changes (95% CI) from baseline compared with control. This trial recorded the effect of dapagliflozin on HbA1c, body weight, fasting insulin and fasting glucose. For HbA1c, this trial showed a mean decline of approximately 0.1% in all active groups (MD=-0.1, SD = 0.01). Moreover, a reduction in body weight of approximately 1 kg was observed on the group of patients treated with dapagliflozin (MD=-1.1 kg, SD = 0.14). No significant effect was noted in fasting insulin and fasting plasma glucose (MD=-0.3, SD = 0.03). Due to the nature in which the data was reported in, with no exact values for the control group, this study was excluded from the SPSS analysis of its respective relevant outcomes.

**Figure 3:**
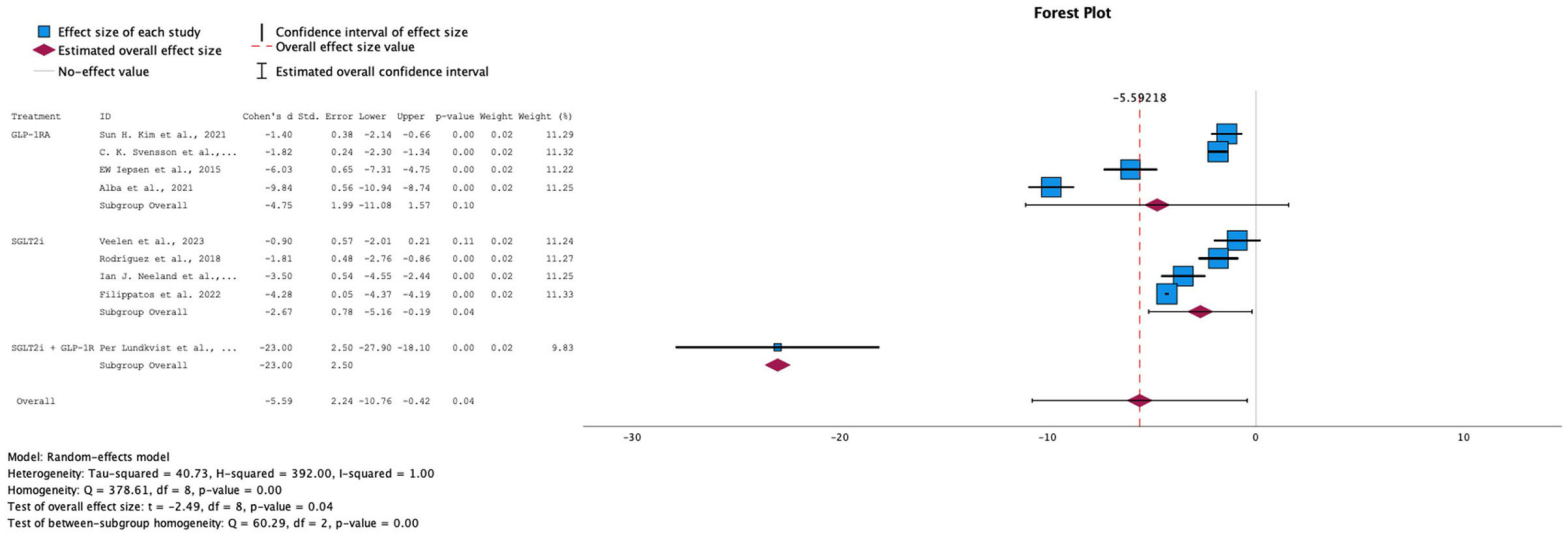
Forest plot of mean difference in body weight changes from baseline in patients receiving treatment compared to placebo. Size of blue box indicates study weight. Red diamond indicates size of estimated overall effect.

Results from the study by Inzuchhi et al. investigating effects of dapagliflozin (13) on incidence of T2DM - as a prespecified exploratory endpoint from the DAPA-HF trial results - reported a significant effect on diabetes onset. Their analysis concluded that Dapagliflozin decreases the risk of incidence by 32%, with the treatment group reporting a rate of onset at 3.4 ± 0.01 per 100 patient years (4.9% incidence) vs 5.0 ± 0.01 per 100 patient years in the placebo group (7.1% incidence). This difference was statistically significant with a p-value of 0.01. While HbA1c was not an outcome measure in itself, it was utilized as a diagnostic tool for the primary outcome. After 8 months of treatment, the study concluded that treatment with dapagliflozin yielded a -0.04% placebo-corrected difference in HbA1c, overall decreasing levels (see **Figure 3**). However, this decrease in HbA1c varied slightly based on the baseline glycemic status of patients, a point which will be further elaborated in our discussion.

Another study reported the effect of dapagliflozin (14) compared to control on many variables. Starting with the HbA1c, the reported values did not show any significance (MD = 0%, SD = 0.6, p-value=0.76). As for the body weight, it reported a decrease compared to placebo (MD=-0.6 Kg, SD = 7.5), however the values were not statistically significant (p-value=0.11). The study reported that fasting insulin values also went down after treatment vs placebo (MD=-4.3, SD = 1.00), however those results were also not statistically significant (p-val= 0.54). Results for fasting plasma glucose were also similar, being not statistically significant (MD=-0.2, SD = 0.5, p-value=0.11).

A study conducted by Anker et al. (15) assessed whether findings from the EMPEROR-Reduced Trial could be stratified further based on glycemic status of participants. One of the secondary outcomes they studied was the time of onset of Diabetes Mellitus in pre-diabetic patients and how it was affected with the administration of empagliflozin vs placebo. The results demonstrated that empagliflozin significantly reduced the incidence of diabetes. In the intervention group, there was an average of 9.31 patients with events per 100 patient-years at risk (SD = 1.1), compared to 10.62 patients (SD = 1.19) in the control group. Statistical analysis showed a Cohen’s d of -1.14, indicating a significant reduction in diabetes incidence due to the treatment, with a p-value of <0. 001. However, it also showed that empagliflozin only lowered HbA1c in diabetic patients and showed no effect in pre-diabetic or normoglycemic patients. As for body weight, SGLT-2i caused a significant reduction in weight compared to placebo. Data for HbA1c and body weight were not made available and thus this study was excluded from our SPSS analysis for these outcomes.

The study by Sun H. Kim et al. (16), which compared the effects of liraglutide and placebo over 14 weeks, found no significant difference in fasting glucose levels between the two groups (MD = -4.02, SD = 1.30, p-value = 0.77). This suggests that liraglutide has minimal impact on reducing fasting glucose levels. Similarly, fasting insulin levels showed no significant reduction with the medication (MD = -10.50, SD = 0.14, p-value = 0.73), indicating its limited effect in lowering fasting insulin. However, body weight demonstrated a significant decrease following the use of the medication, with a statistically significant result (MD = -6.10, SD = 1.90, p-value < 0.05).

A study by C. K. Svensson et al. (17) investigated the effects of a 16-week course of liraglutide versus placebo on body weight and glucometabolic parameters in prediabetic patients with schizophrenia-spectrum disorders on stable clozapine or olanzapine therapy. The results showed a significant reduction in HbA1c levels in the liraglutide group compared to placebo (MD = -2.90, SD = 2.1, p-value < 0.05), confirming its effectiveness in lowering HbA1c. Furthermore, regarding fasting plasma glucose, no significant results were noted as p-value was greater than 0.05 hence it showed that the difference was very little between the 2 groups (MD= -1,13, SD = 1.02, p-value= 0.50). Additionally, a significant reduction in body weight was observed in the treated group (MD = -2.40, SD = 1.10, p-value < 0.05), confirming that liraglutide effectively aids in weight loss.

In the study of EW Iepsen et al. (18) a double-blind, randomized clinical trial with a placebo control group using liraglutide as the treatment for 8 weeks; body weight showed a difference between both groups (MD = 0.40, SD = 0.29, p-value = 0.00). It showed that body weight is decreased as we use liraglutide and it is statistically significant as the p-value obtained is less than 0.05 which supports the claim above.

Results from the study of Alba et al. (19) showed a slight reduction in HbA1c levels compared to the placebo group and it was statistically significant (MD= -0.24, SD = 0.03, p-value<0.01). Additionally, fasting insulin levels were compared, and it showed a statistically significant decrease in the fasting insulin levels with respect to the placebo group (MD= -0.02, SD = 1.37, p-value< 0.01) as the p-value is less than 0.05. Furthermore, fasting plasma glucose levels showed a slight but statistically significant decrease in the liraglutide group compared to the placebo group (MD = -5.70, SD = 1.50, p-value < 0.05). Similarly, body weight comparisons revealed that weight loss goals were achieved with liraglutide, with a statistically significant difference compared to placebo (MD = -7.50, SD = 0.50, p-value < 0.05).

Results from Per Ludkvist et al. (20) studied the effect of dual therapy with dapagliflozin and exenatide on body weight, body composition, glycemic variables and systolic blood pressure (SBP) in obese adults without diabetes. After 24 weeks, for dapagliflozin/exenatide versus placebo: the difference in body weight change was −4.13 kg (95% confidence interval −6.44, −1.81; p-value <.001). Dapagliflozin/exenatide significantly reduced HbA1c and fasting plasma glucose (FPG) compared to placebo over 24 weeks, with mean differences of −2.3 mmol/mol (−0.21%) and −0.66 mmol/L, respectively (p-value<0.001). Another study investigated the effects of dapagliflozin vs placebo (21). Starting with HbA1c levels, this trial showed a slight drop after treatment compared to placebo (MD = -0.5%, SD = 0.1), which was statistically significant (p-value<0.001). It also showed a decrease in body weight in treated subjects (MD = -1.0 kg, SD = 0.0). Finally, for the fasting plasma glucose, it showed a decrease in treated subjects (MD = -0.5, SD = 0.2) which was also statistically significant (p-value<0.001).

Another trial by Rodriguez et al. showed that after dapagliflozin administration (22), a remarkable decrease in body weight was noticed (MD = -3kg, SD = 1.4) which was statistically significant (p-value<0.01). Also, a considerable decrease in fasting glucose levels was noted (MD = -0.8mmol/l, SD = 0.1, p-value<0.01). Lastly, it reported that dapagliflozin can play a role in slightly decreasing HbA1C levels (MD = -0.1%, SD = 0.00). A decrease in fasting insulin was also noted, but it was not statistically significant (MD = -4.5pmol/l, SD = 6.3, p-value = 0.9).

Finally, the last study done by Neeland et al. on empagliflozin (23) compared to a placebo group showed an HbA1C drop compared to the control group (MD = -0.1%, SD = 0.02%, p-value<0.01). Additionally, a significant reduction in body weight was observed (MD = -5.5 kg, SD = 2.66, p-value < 0.01). Finally, the study also demonstrated a statistically significant decrease in fasting glucose levels in the treatment group compared to the placebo group (MD = -1.6, SD = 0.4, p-value < 0.01).

### 3.4 Results of synthesis

The majority of the studies included in this meta-analysis were double blinded randomized controlled trials (13/14). This led to a low risk of selection bias in practically most of our studies. Baseline differences between the treatment and the control group were always present implicating an effective randomization process. Performance bias was reduced in 13/14 of the studies since participants were unaware of what drug they were taking in the beginning of the study.

Concerning the results, several outcomes were found to be reduced in both SGLT-2 inhibitors and GLP-1 receptor agonists studies. In fact, body weight reduction was highlighted in both drugs compared to the control groups (MD = -5.5kg, SD = 2.66, p-value<0.01), as well as fasting blood glucose (MD = -1.6mmol/L, SD = 0.4, p-value <0.01). HbA1c was targeted in 9 studies with 8 of them showing reductions compared to the placebo group and one study (14) showed no change after treatment. Furthermore, studies on GLP-1 receptor agonists demonstrated a more consistent reduction in HbA1c levels compared to those on SGLT-2 inhibitors. In fact, two studies on SGLT-2i reported no to minimal reduction in HbA1c (-0% and -0.07% respectively), while the rest showed more substantial decreases, in particular one GLP-1 RA study with a statistically significant decrease of -2.90%. However, it is important to note that more studies focused on SGLT-2i individually than on GLP-1 RA. Five studies on SGLT-2 inhibitors demonstrated a reduction in the onset of diabetes mellitus. Among these, three studies specifically investigated and reported a decrease in the number of patients with events per 100 patient-years at risk, focusing solely on the treatment group receiving SGLT-2 inhibitors.

All of these findings highlight the various benefits of SGLT-2 inhibitors and GLP-1 agonists in delaying type 2 diabetes mellitus onset, reducing HbA1c levels, as well as decreasing fasting blood glucose.

→ Effect on body weight

Of the 14 RCTs analyzed for our study, 9 papers included measurements on the effect of SGLT-2i, GLP-1 RA, or their combination, on body weight as compared to placebo. Random-effects model was applied due to the high heterogeneity among the studies (I-squared = 100%). The overall effect size showed that all treatments were successful in decreasing body weight in pre-diabetic patients, with a statistically significant difference (p-value = 0.04). When comparing individual treatment groups, SGLT-2i demonstrated a statistically significant standardized mean difference (SMD) of -2.67. In contrast, the GLP-1 RA intervention, although it showed a larger SMD (-4.75), did not reach statistical significance, with a p-value of 0.10. Interestingly, when both SGLT-2i + GLP-1 RA were combined, the study showed that this intervention yielded the best results (SMD: -23, 95% CI: [-27.9, -18.10]), which suggests that the combination of these drugs shows potential in being the better option in terms of weight loss. Although the effect looks promising, it is based on only one small study; the estimate is imprecise and should be interpreted as preliminary and hypothesis-generating. This encourages more studies to be done on the combination of GLP-1 RA + SGLT-2i with a higher number of participants to yield more reliable results.

→ Effect on HbA1c levels

**Figure 4:**
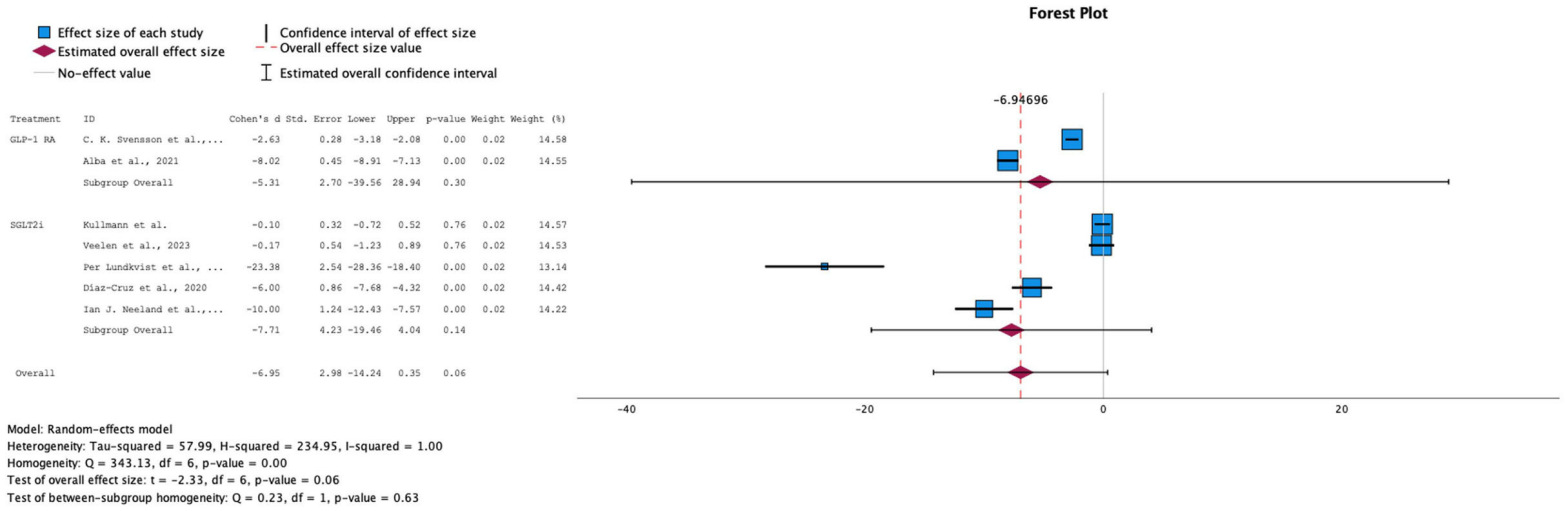
Forest plot of mean difference in HbA1c (%) changes from baseline in patients receiving treatment compared to placebo. Size of blue box indicates study weight. Red diamond indicates size of estimated overall effect.

Both GLP-1 RA and SGLT-2i, individually and in combination, resulted in a decrease in HbA1c levels, with a standardized mean difference of -6.95 (95% CI: [-14.24, 2.98], p-value= 0.06) for the overall effect size (see **Figure 4**). 9 out of 14 of our studies included data on HbA1c as an outcome. However, one study (22) was excluded from the analysis because the calculated difference of the standard deviation (SD) between baseline levels and follow-up values was equal to 0, and another (12) due to the values for the interventional group being reported as changes from baseline as compared to the control group rather than as two sets of data. The 7 remaining studies showed a high level of heterogeneity (I-squared: 100%), prompting the use of a random-effects model. Overall, the studies demonstrated a general trend of decreasing levels, with a statistically significant overall p-value of 0.06. However, the subgroup analyses for each drug individually showed wide confidence intervals.

→ Effect on the Diabetes Onset

**Figure 5:**
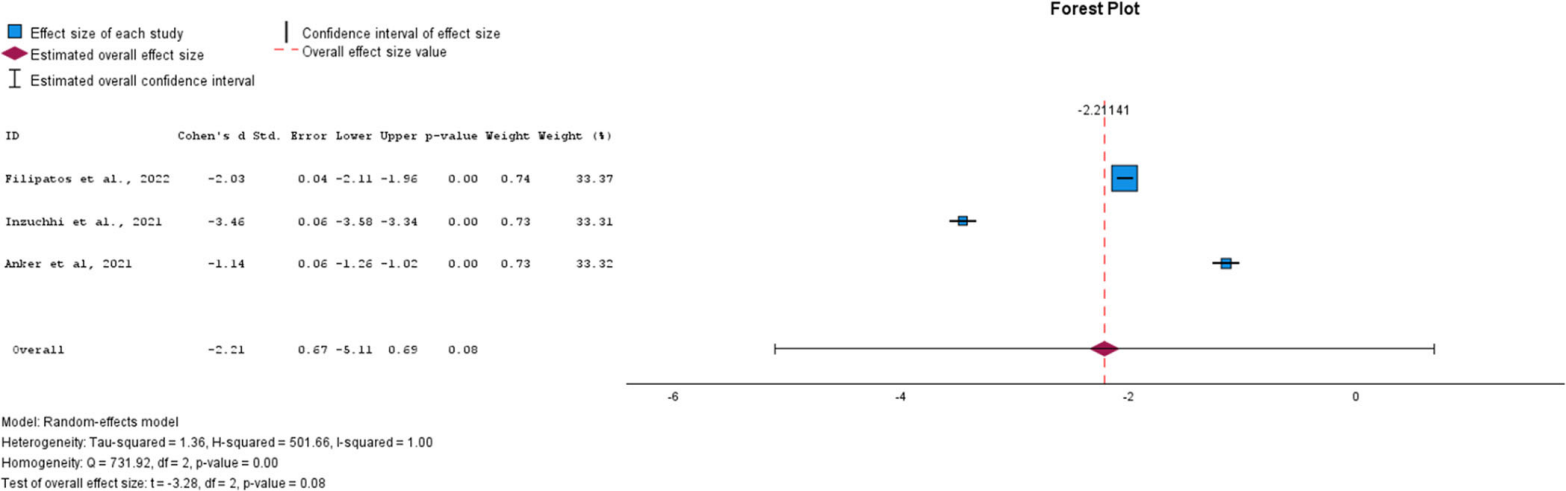
Forest plot of mean difference of diabetes onset from baseline in patients receiving treatment compared to placebo. Size of blue box indicates study weight. Red diamond indicates size of estimated overall effect.

For the diabetes onset outcome, which measured the number of events per 100 patient-years at risk following treatment, data were available only for SGLT-2i compared to the control group. All three studies showed a reduction in the number of diabetes onset cases, suggesting that SGLT-2i treatment demonstrates promising potential in delaying the onset of diabetes when started during the prediabetes stage. However, the overall effect was not statistically significant (p = 0.08, SMD = -2.21, 95% CI: [-5.11, 0.69]; see **Figure 5**). This lack of significance may be due to variability in effect sizes among the studies. The studies exhibited high heterogeneity (I² = 100%), necessitating the use of a random-effects model.

→ Effect on Fasting insulin levels

**Figure 6:**
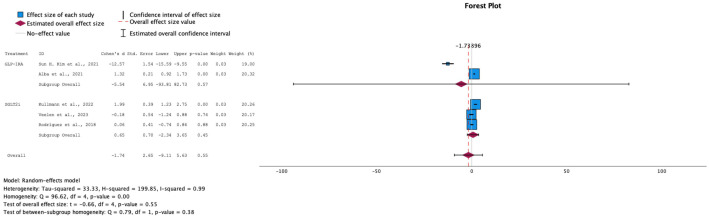
Forest plot of mean difference of fasting insulin changes from baseline in patients receiving treatment compared to placebo. Size of blue box indicates study weight. Red diamond indicates size of estimated overall effect.

The trend in insulin levels, though leaning toward a negative change, did not show a significant effect, with an overall SMD of -1.74 (95% CI: [-6.84, 3.37]) and a p-value of 0.55. GLP-1 RA primarily drove this negative trend, while the overall effect size for SGLT-2i remained close to null (see **Figure 6**). This outcome aligns with the expected differences in the mechanisms of action of the two drug classes. Due to the high heterogeneity among the studies (I² = 99%), a random-effects model was applied. These findings highlight the need for further research into the effects of these drugs on insulin levels in prediabetic patients.

→ Effect on Fasting plasma glucose levels

**Figure 7:**
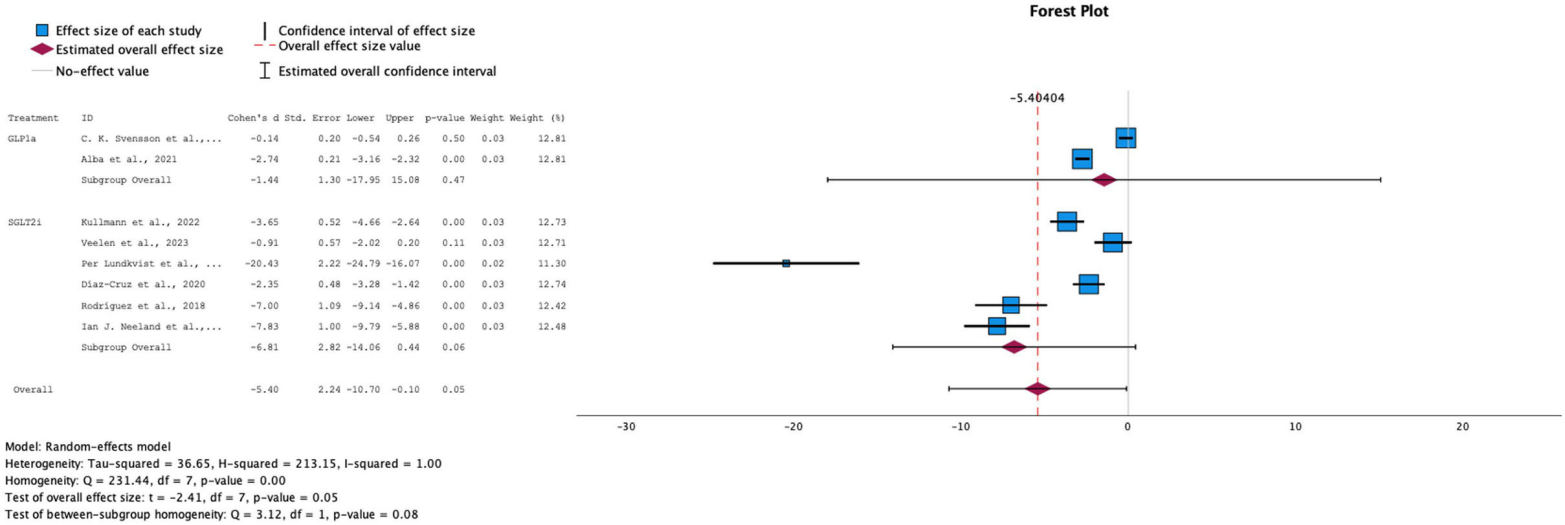
Forest plot of mean difference of fasting plasma glucose changes from baseline in patients receiving treatment compared to placebo. Size of blue box indicates study weight. Red diamond indicates size of estimated overall effect.

For fasting plasma glucose levels, both interventions showed a decrease across all studies, with a statistically significant overall effect (SMD = -5.40, 95% CI: [-10.70, -2.24]) and a p-value of 0.05 (see **Figure 7**). However, the eight studies that reported fasting glucose measures exhibited high heterogeneity (I² = 100%), necessitating the use of a random-effects model for this analysis.

### 3.5 Reporting biases

Publication biases were assessed using the funnel plot and Egger’s regression test for all our outcomes. HbA1c plot showed symmetry with no significant biases. On the opposite hand, plots for fasting insulin were asymmetric when analyzed for both subgroups simultaneously, highlighting the presence of context-specific biases, but when studied separately, plots showed no biases since they were symmetrical. For fasting glucose levels, asymmetry was seen when subgroups were analyzed together. Body weight was significantly decreased in both drugs, noting symmetry in plots showing no biases. Also, the small number of participants in some studies and the limited number of outcomes reported in some studies could raise some concerns for publication bias.

### 3.6 Certainty of assessment

The certainty of assessment was evaluated using the GRADE approach for each outcome of our meta-analysis. Grades were assigned as high, moderate, low or very low while taking into consideration several factors such as publication bias, limitations and imprecision. The major outcome, included in most of the studies; HbA1c, a grade of high was assigned due to the low risk of publication bias. Although HbA1c was initially graded as ‘High’ owing to the predominance of randomized, double-blind RCTs and a low risk of publication bias, the very high between-study heterogeneity (I² ≈ 99–100%) and indirectness of several populations temper the certainty and warrant cautious interpretation of the pooled estimate. All other outcomes, such as body weight and fasting glucose, were assigned grades ranging from moderate to low due to the presence of asymmetry in their plots and the different limitations encountered in every study used such as the limited participants number and the short period of intervention.

## 4 Discussion

Type 2 diabetes mellitus (T2DM) is a serious health concern faced all over the globe, with its prevalence exhibiting an increasing trend over the years and is projected to continue to do so. As of 2021, and according to the International Diabetes Federation (IDF) Diabetes Atlas and the Global Burden of Disease, T2DM showed a global prevalence of 537 million adults aged 20–79 years old (24) - what equates to 1 in 10 (25). Compared to the 462 million individuals affected in 2017 (26), this is a considerable and alarming rise.

Over the course of time, diabetes can lead to multiple comorbidities. It is the leading cause of nephropathy and end-stage kidney disease, neuropathy, retinopathy and adult-onset blindness, non-traumatic amputation, and contributes greatly to heart disease and cardiovascular morbidity and mortality (27). In 2021, it was responsible for 6.7 million deaths (24). This highlights concerns about the costs and financial burden of treatment and management on both individuals and the healthcare systems. With the rising number of people at risk of developing T2DM, and the severe complications related to the disease, the priority moving forward should be identifying effective strategies to prevent their progression to diabetes. In this regard, our results show a promising potential in directing the next steps towards finding the best approach.

This meta-analysis showed that when initiated at the prediabetic stage, interventions with medications, specifically SGLT-2 inhibitors and GLP-1 receptor agonists, show promising evidence in delaying the progression of prediabetes into diabetes.

Data were extracted from 14 randomized controlled trials for this meta-analysis, which specifically explored effects of SGLT-2i and/or GLP-1 RA on pre and/or non-diabetic patients exclusively. Of these, 8 studies reported a downward trend in HbA1c levels due to these medical interventions, with the majority showing statistically significant results. However, a range in the magnitude of this decrease was seen across the studies, with the highest drop in levels being reported to be statistically significant at - 2.9% from baseline. This may be attributed to the difference in effect that treatment may have on HbA1c in individuals based on their baseline glycemic status. This was demonstrated by Inzuchhi et al, where they noted that when separating the non-diabetic group of patients further down to prediabetic (HbA1c 5.7-6.4%) and normoglycemic (<5.7%) patients, the treatment showed a slight variation in its effect on HbA1c in the respective groups, where in prediabetics it yielded a placebo-corrected reduction of -0.04%, compared to placebo-corrected reduction of +0.05% in normoglycemics (13). Despite these variations in the magnitude of the effect, there was another outcome within our RCTs that aligned with this overall negative trend. 3 studies investigated time to onset of Diabetes Mellitus from randomization till completion of the trial as a secondary outcome. All 3 showed that, compared to control groups treated with placebo, medical interventions with SGLT-2i in specific (as no data was available for GLP-1 RA on this outcome) delayed the progression to and onset of diabetes, where the incidence of new diabetes diagnosis at the end of the trial showed a notable decrease as compared to placebo. Two additional studies, one using GLP-1 RA and SGLT-2i and the other using only SGLT-2i, demonstrated a reduction in the proportion of participants meeting the diagnostic criteria for prediabetes after treatment. These studies also assessed markers of prediabetes, including impaired fasting glucose (IFG), impaired glucose tolerance (IGT), and HbA1c levels. Following treatment, participants’ values for these markers significantly improved, falling below the diagnostic thresholds for prediabetes (20, 21).

To further solidify this claim, we interpreted overlapping data among the trials on other risk factors and serum studies that may contribute to or be used as markers for diabetes onset. A statistically significant overall decrease was noted in the fasting plasma glucose levels post medical intervention at the prediabetic stage (SMD: -5.40, 95% CI: [-10.70, -0.10], p-value: 0.05). SGLT-2i in particular showed a more prominent effect with a standardized mean difference of -6.81 (95% CI: [-14.06, 0.44]; p-value: 0.06) as compared to GLP-1 RA that sat at -1.44 (95% CI: [-17.95, 15.08]; p-value: 0.47). This in turn explains the overall negative effect these same interventions exhibited on fasting insulin levels, however, this change in effect was quite small, and was statistically insignificant with a p-value of 0.55. It is important to highlight that while SGLT-2 inhibitors did not show a significant effect and remained at the neutral line, the studies on GLP-1 receptor agonists were the primary contributors to the observed decrease in insulin levels. This is contrary to expectations, as the mechanism of action of GLP-1 agonists typically involves increasing insulin secretion to help lower blood glucose levels. However, this discrepancy among studies in the effect on insulin, particularly with GLP-1 agonists, may be due to simultaneous effects that GLP-1 RA has on body weight loss, which in turn exhibits pleiotropic effects in improving insulin sensitivity and decreasing insulin secretion (16).

This brings us to the discussion of our findings on body weight effects. As previously mentioned, T2DM is caused by multi-organ insulin resistance, followed by a decline in pancreatic beta-cell secretory function. Studies have shown that accumulation of excess body fat and the progressive increase in body mass index (BMI) - which is also an index of adiposity - is associated with a progressive increase in the risk of developing type 2 diabetes (28). Individuals with obesity exhibit elevated basal and postprandial plasma insulin levels, primarily due to an increased secretion rate from beta cells. This results from beta cell hyperplasia, which leads to approximately a 50% increase in pancreatic beta cell mass (28), as well as a reduction in insulin clearance. This initially aids in overcoming the insulin resistance in obese patients and allows blood glucose levels to remain normal. On the other hand, the sensitivity to insulin is largely affected by adipose tissue metabolic function and interactive biologic processes, which are found to be abnormal in obesity. This includes the secretion of proinflammatory proteins and exosomes from adipose cells that promote organ resistance to insulin, as well as an inverse relationship between percent body fat and adiponectin secretion - an insulin sensitizing agent - where it is decreased in obese individuals. Over time, due to mechanisms that remain unclear, pancreatic beta cells undergo apoptosis as type 2 diabetes mellitus (T2DM) and impaired fasting glucose progress. This leads to patients with elevated BMI losing their ability to compensate for insulin resistance (28). Therefore, loss of weight can have significant effects on improving multiorgan insulin sensitivity, as shown by many studies in the literature. Results from our meta-analysis indicates that, while GLP-1 receptor agonists (GLP-1 RA) and SGLT-2 inhibitors (SGLT-2i) are well-established for promoting weight loss, their combination demonstrates significant potential as a superior option, offering an additive effect in reducing body weight compared to using either drug alone (SMD: -23.00, 95% CI [-27.9, -18.10], p-value: 0.00). Furthermore, while GLP-1 RA showed a higher overall reduction in body weight compared to SGLT-2i, the latter showed a statistically significant result with a p-value of 0.04 (former showing 0.10). It is important to note that each subgroup consisted of 4 studies, and all 8 studies showed consistently similar effect sizes across the board.

Finally, this meta-analysis opens new horizons for further investigations on the long-term efficacy of the medications studied. Our study shows considerable effects on lowering HbA1c levels, body weight, and expanding the diabetes mellitus time of onset and other parameters, proving that intervention at the prediabetic stage can successfully delay the onset of T2DM. However, these findings cannot be extrapolated to complete prevention of diabetes progression, especially since it did not include any follow-up data for the patients past the estimated duration of the trials. As previously discussed, the gradually worsening ophthalmologic, neurologic, and nephrotic complications associated with diabetes are key factors that make it a serious health concern in today’s world.

## 5 Limitations

Several limitations should be considered in our study. Some of the studies have a small number of participants which raises concerns for statistical errors and inability to draw conclusions for the whole population. On another note, some studies had either a short intervention period or a short follow-up period, both of which can lead to inability to fully know both the therapeutic and side effects of the drugs used. In addition, this paper includes a mix of articles that don’t report the exact same outcomes, making it difficult for a comprehensive comparison between the different drugs in question. In a distinct regard, population differs from study to study; for example, a study (17) was held on a schizophrenic population thus making generalization of its outcomes not reasonable. Finally, the mix in the studies’ designs (parallel, cross-over and multi-arm) used in this meta-analysis makes the risk of bias much higher in comparison to it being done using only double-blinded studies. All these factors contributed to the high I-squared and heterogeneity in the meta-analysis of our studies. It is important to note that the very high heterogeneity observed across key outcomes (I² = 99–100%) reflects the diversity of study populations, interventions, and designs. Although robust random-effects methods were applied to provide conservative pooled estimates, subgroup or leave-one-out analyses were not feasible given the limited number of homogeneous trials.

Future research should prioritize conducting double-blinded studies, focusing on a consistent population, evaluating the same outcomes, implementing longer treatment durations, and ensuring appropriate patient follow-up.

## 6 Conclusion

In conclusion, our meta-analysis has shown that the initiation of treatment at the prediabetic stage, whether with either GLP-1 RA or SGLT-2i, shows promising evidence in delaying the onset of type 2 diabetes diagnosis. This, however, cannot be extrapolated to complete prevention as further studies need to be done with longer follow-up periods to assess if these medications can prevent the onset of diabetes, and the duration and dose of medication needed to do so. Furthermore, in terms of comparing the two interventions, SGLT-2i seems to show more favorable evidence in delaying the onset of DM as well as decreasing HbA1c and fasting plasma glucose to a higher extent. However, it is important to note that we consistently found more data available and studies done on SGLT-2i compared to GLP-1 RA across all outcomes, especially DM onset, and even less data on the combination of the two. This prompts the need for more studies to investigate the matter in more detail.

## Data Availability

The original contributions presented in the study are included in the article/**Supplementary Material**. Further inquiries can be directed to the corresponding authors.
